# Voluntary Wheel Running Did Not Alter Gene Expression in 5xfad Mice, but in Wild-Type Animals Exclusively after One-Day of Physical Activity

**DOI:** 10.3390/cells10030693

**Published:** 2021-03-20

**Authors:** Anna Wierczeiko, Lena Gammel, Konstantin Radyushkin, Vu Thu Thuy Nguyen, Hristo Todorov, Susanne Gerber, Kristina Endres

**Affiliations:** 1 Working Group Computational Systems Genetics (CSG), Institute of Human Genetics, University Medical Center, Johannes Gutenberg University, 55131 Mainz, Germany; Anna.Wierczeiko@lir-mainz.de (A.W.); hristo.todorov@uni-mainz.de (H.T.); 2 Working Group Mouse Behavioral Unit (MBU), Leibniz Institute for Resilience Research (LIR), 55122 Mainz, Germany; radyushkin@uni-mainz.de; 3 Working Group Healthy Aging and Neurodegeneration, Department of Psychiatry and Psychotherapy, University Medical Center, Johannes Gutenberg University, 55131 Mainz, Germany; lenagammel@googlemail.com (L.G.); VuThuThuy.Nguyen@unimedizin-mainz.de (V.T.T.N.)

**Keywords:** physical activity, Alzheimer’s disease, 5xFAD, chronic, acute, wheel running

## Abstract

Physical activity is considered a promising preventive intervention to reduce the risk of developing Alzheimer’s disease (AD). However, the positive effect of therapeutic administration of physical activity has not been proven conclusively yet, likely due to confounding factors such as varying activity regimens and life or disease stages. To examine the impact of different routines of physical activity in the early disease stages, we subjected young 5xFAD and wild-type mice to 1-day (acute) and 30-day (chronic) voluntary wheel running and compared them with age-matched sedentary controls. We observed a significant increase in brain lactate levels in acutely trained 5xFAD mice relative to all other experimental groups. Subsequent brain RNA-seq analysis did not reveal major differences in transcriptomic regulation between training durations in 5xFAD mice. In contrast, acute training yielded substantial gene expression changes in wild-type animals relative to their chronically trained and sedentary counterparts. The comparison of 5xFAD and wild-type mice showed the highest transcriptional differences in the chronic and sedentary groups, whereas acute training was associated with much fewer differentially expressed genes. In conclusion, our results suggest that different training durations did not affect the global transcriptome of 3-month-old 5xFAD mice, whereas acute running seemed to induce a similar transcriptional stress state in wild-type animals as already known for 5xFAD mice.

## 1. Introduction

As the population ages, the number of people with degenerative diseases is simultaneously increasing. Dementia is the fifth most frequent cause of death worldwide, with Alzheimer’s disease (AD) as the most prevalent type of dementia in the elderly [1]. AD is characterized by a progressive decline in memory functions, behavioral impairments, and the loss of social skills, which means that quality of life begins to deteriorate well before death [2,3]. Key neuropathological features of this devastating disorder include the accumulation of extracellular β-amyloid (Aβ) plaques, intracellular neurofibrillary tangles (NFT) composed of hyperphosphorylated tau proteins, and region-specific synaptic as well as cellular degeneration, together with an increase in inflammation and oxidative stress [4,5,6,7]. In addition, metabolic dysfunctions such as abnormal glucose uptake and brain insulin resistance, likely increasing degeneration and cognitive impairment, have also been described in AD patients [8]. Besides the known pathological hallmarks, the leading cause of progressive brain atrophy is still unknown, explaining the lack of successful AD treatments. It became, however, generally accepted that the underlying mechanisms are polyfactorial and depend on the complex interplay of multiple (partly unknown) genetic and non-genetic variables [9,10,11,12]. Furthermore, it is widely recognized that AD already seems to develop decades before clinical symptoms occur [13,14,15,16]. Since prophylactic pharmacological therapies in advance of a possible disease outbreak might be ethically not reasonable or hampered by lack of a long-term compliance, lifestyle-modifying interventions, and preventions such as diet and physical activity have become increasingly attractive in the neurodegenerative research field.

Physical activity (e.g., aerobic exercise or resistance training) has already been associated with several beneficial effects that reduce the risk of developing AD in both, humans and animal models [17,18,19,20]. The duration of physical activity being studied can be divided into acute, one single bout of physical activity, and chronic, consistent activity over an extended time period [21]. Several studies suggested that regular training can improve reaction time, increase the size of the hippocampus (the brain region mainly responsible for learning and memory), and delay age-related memory impairments as well as the overall cognitive decline in older individuals with and without mild-cognitive impairment (MCI) or early AD [18,22,23,24]. Even after just one exercise session, short- and long-term memory and immune cell function were presumably stimulated in normal aging and MCI cohorts [25,26,27]. In transgenic rodent AD models, chronically trained animals exhibited a reduction in Aβ deposition and tau phosphorylation, anti-inflammatory modifications, improved brain oxygenation, and general cognitive improvements as compared to sedentary littermates [13,28,29,30,31,32,33]. Furthermore, another study showed that only three days of voluntary wheel running was associated with enhanced neurogenesis in rats [34].

However, there is still insufficient evidence that physical activity provides an effective method of fighting AD, as there are also several studies that have not observed a positive link between physical activity and brain function improvement [35,36,37,38,39]. Recently, Hansson et al. conducted a large longitudinal study over 20 years that indicated no reduction in the frequency of developing AD by comparing exercising participants (*n* = 197,685) with sedentary individuals (*n* = 197,684) [37]. The same results were obtained in a subsequent study on chronically trained AD model mice (5xFAD) [37]. The evaluation of neuroinflammation and non-cognitive parameters in voluntarily running young 5xFAD mice, in which no pathological features had yet developed, also showed no changes compared to sedentary mice. Moreover, signs of disease acceleration could even be identified within the trained transgenic group [38]. The inconsistency of physical activity effects on cognitive function and AD could likely be explained by different starting points such as age or disease state, duration (e.g., acute or chronic), and activity types [18].

On the neurochemical level, specific neurotransmitters, neuromodulators, and lactate concentrations were shown to increase after short and long-term exercise [40]. Furthermore, some genes and proteins that are predominantly involved in neurogenesis were suggested to be differentially expressed after both, acute and regular physical activity, in humans and in animal models, with brain-derived neurotrophic factor (BDNF) being the most prominent [34,41,42]. The global transcriptional response to different types of physical activity in AD has rarely been investigated. To better understand the molecular biology behind physical activity in AD, we examined in this study the blood and brain lactate levels as well as the transcriptomes of 3-month-old transgenic 5xFAD and wild-type mice after one day (acute) or four weeks (chronic) voluntary wheel running. Comparing the gene expression of 5xFAD mice at an age at which the pathology is not severely manifested with age-matched controls could provide further insights into fundamental molecular mechanisms of short- and long-term exercise in both AD and healthy individuals.

## 2. Materials and Methods

### 2.1. Animals

Male B6SJL-Tg(APPSwFlLon, PSEN1*M146L*L286V)6799Vas/Mmjax (5xFAD) mice (Jackson Lab, Bar Harbor, Maine, USA) were crossbred with female C57BL/6J mice and maintained as heterozygous transgenics on the C57BL/6J background. The animals were single-caged in type II cages with a 12-h day/night cycle. Food and water were available ad libitum. The non-transgenic littermates were used as the control. All procedures were performed in accordance with the European Communities Council Directive regarding care and use of animals for experimental procedures and were approved by local authorities (Landesuntersuchungsamt Rheinland-Pfalz; approval number G 14-1-087). Mice were randomly assigned to three groups: untrained (sedentary), access to a saucer wheel (15-cm diameter; med associates Inc.) for 30 days, or access to a saucer wheel for 18 h before sacrifice. The saucer wheel contained a WLAN connection to a computer for measuring running counts. Food consumption and body weight were assessed regularly.

### 2.2. Tissue Preparation

Mice were sacrificed after isoflurane anesthesia, and truncal blood was collected. Blood glucose was measured immediately from two independent droplets with an AccuCheck mobile device (Roche). Heart, muscle, abdominal fat, and the left brain-hemisphere were dissected and shock-frozen in liquid nitrogen with subsequent storage at −80 °C. The right hemisphere was cut into small cubicles and incubated at Room Temperature for 20 min in RNAlater (Qiagen, Hilden, Germany) before being stored at −80 °C. Serum was obtained from truncal blood by two-times centrifugation after clotting and stored at −80 °C.

### 2.3. Lactate Measurement

Brain samples were homogenized with a Tissue Lyzer, and stainless-steel beads (Qiagen, Hilden, Germany) in 600 µL 0.5M KH_2_PO_4_-buffer supplemented with protease inhibitor (cOmplete, Roche) pH7 for 5min at 50 Hz. 20 µL of the homogenate and 2.5 µL of serum were subjected to the enzymatic assay.

Lactate was measured following the protocol from Lin and colleagues [43]. In brief, the assay uses coupling of lactate-oxidase and peroxidase for the conversion of 2,2′-azino-bis (3-ethylbenzothiazoline-6-sulfonic acid) (ABTS, Sigma Aldrich, Steinheim, Germany). As a standard, lithium-lactate was used, and the absorbance of the chromogenic product was measured at 405 nm. The protein content of all samples was assessed with Roti-Nanoquant (Roth) and used for normalization.

### 2.4. RNA-Seq Library Preparation and Sequencing

Next-generation sequencing library preparation was performed with Illumina’s TruSeq stranded mRNA LT Sample Prep Kit following Illumina’s standard protocol (Part # 15,031,047 Rev, E). The libraries were prepared with a starting amount of 302 ng and amplified in 12 PCR cycles, profiled in a DNA 1000 Chip on a 2100 Bioanalyzer (Agilent Technologies) and quantified using the Qubit dsDNA HS Assay Kit, in a Qubit 2,0 Fluorometer (Life technologies). All 24 samples were pooled in equimolar ratio and sequenced on 1 NextSeq 500 Highoutput FC, PE for 2 × 42 cycles plus 7 cycles for the index read.

### 2.5. RNA-Seq Analysis

After merging five technical replicates and down-sampling one sequencing file, the reads were trimmed using BBDuk (version 38.06) [44]. For the differential expression analysis, the trimmed reads were mapped to the UCSC reference genome of *Mus musculus* obtained through Illumina iGenomes (version mm10, latest release in May 2012) [45], using the splice-aware mapper STAR (version 2.7.0d, default options of 2-pass mapping) [46]. The aligned reads were then counted per gene and sample (based on the mm10 UCSC annotation file from iGenomes) using FeatureCounts provided by SubRead (version 1.6.2, default options for single-end reads) [47].

The differential expression analysis (DEA) was done in R (version 4.0.3) and RStudio (version 1.3.1073) using the R package DESeq2 (version 1.30.0) [48,49,50]. In order to reduce the impact of extreme expressions, three genes, Psen1, App, and Thy1, which are known to be highly differentially expressed due to the genotype of 5xFAD mice [51], were removed from the count tables. The normalized gene counts of these marker genes can be found in Supplementary Figure S1. After normalizing the filtered gene counts by the size factors provided by DESeq2, a principal component analysis was performed for all mice and each genotype separately [52]. Additionally, PCA was conducted based on the separate training groups and is shown in the Supplementary Figure S2. The *p*-values of the DEA were adjusted using the Benjamini–Hochberg method, and the cut-off for significant differential regulation was set at adjusted *p*-value < 0.05 [53].

Gene Ontology (GO) term enrichment and Kyoto Encyclopedia of Genes and Genomes (KEGG) pathway enrichment analyses were performed for all subsets of differentially regulated genes using the R package clusterProfiler (version 3.18.0) [54]. Furthermore, the gene subsets were examined for brain cell-type enrichment using the Fisher’s Exact Test implemented in R [55]. The cell-type-associated gene list consisted of five brain cell types, including neurons, astrocytes, oligodendrocytes, microglia, and endothelial cells, and is based on a previously published brain transcription analysis [56,57]. The gene universe (i.e., the gene’s background set for the statistical analysis) was set to the genes considered for the differential expression analysis for all tests. After adjusting the *p*-values of the overrepresentation test using the Benjamini–Hochberg method, the terms with an adjusted *p*-value < 0.05 were considered as significantly enriched.

All plots were created using the R packages ggplot2 (version 3.3.2) and venn (version 1.9) [58,59]. The raw fastq files and the count table were uploaded to Gene Expression Omnibus (GEO) under the accession number GSE164798 (see Data Availability Statement).

## 3. Results

### 3.1. Voluntary Running Wheel Usage in 5xFAD Mice

**Figure 1:**
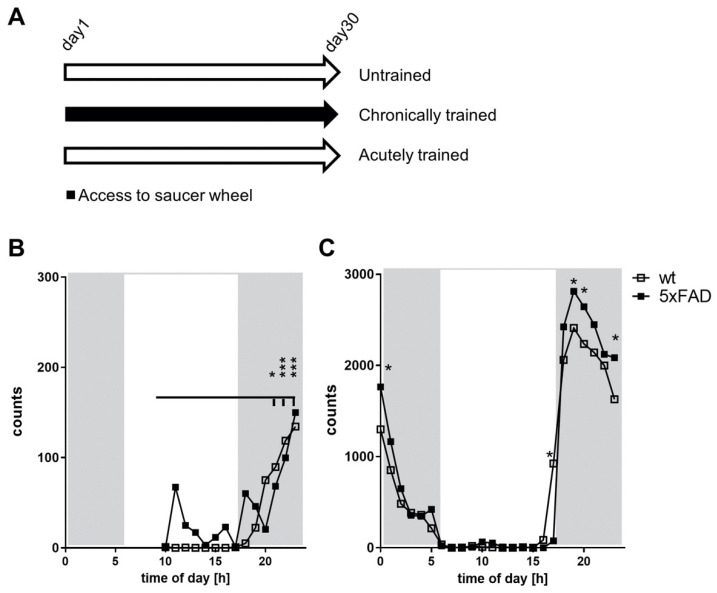
Training groups and the comparison of first night and chronic saucer wheel usage. (**A**) Schematic of the three groups used for the investigation. All animals were single-caged and kept in type II macrolon cages for 30 days. The untrained group had no access to a saucer wheel, the chronically trained group kept the wheel over the whole time period, and the acutely trained mice had access only for the last night. Wheel turning counts per hour were measured for new access to the saucer wheel (**B**) and the chronic usage (**C**). Data are presented as mean of the performance over the 30 days of the chronically trained mice (*n* = 10 for wild-type, *n* = 8 for 5xFAD mice). Error bars are not visualized for clarity of the graph. Statistical analysis: multiple unpaired t-tests (* *p* < 0.05; *** *p* < 0.001).

Physical activity has often been discussed as beneficial in AD or animal models of the disorder. To analyze the potential impact of acute and chronic physical voluntary training, we investigated male mice with the 5xFAD genotype and their wild-type littermates, starting at the age of 2 months. At this age, no behavioral deficits have been reported in the mice; however, deposition of Aβ is observable already at 1.5 months of age [51]. Mice were single-caged and divided into three groups: an untrained group with no access to a saucer wheel, a chronically trained group with full access over the 30 days of the experiment, and the third group with access only within the last night before sacrifice (see Figure 1A). Running activity was assessed by a wireless reporting system: within the first night after adding the saucer wheel, animals of both genotypes quickly adjusted to the new enrichment during the first hours after being exposed to the saucer wheel. This is indicated by a significant count increase in both genotypes starting at 22 p.m. (comparison vs. starting point at 10 a.m., Figure 1B). No apparent differences occurred in the starting phase in both genotypes despite slight but non-significantly higher arousal in 5xFAD mice. Approximately 150 counts for wheel turning per hour were reached at 12 a.m. With prolonged access to the saucer wheel, counts as high as 2800 per hour were reached on average (Figure 1C) with a clear peak in the early dark phase (between 6 and 10 p.m). 5xFAD mice showed a statistically significant elevation of wheel usage at several points of time, resulting in an increased mean running distance (5xFAD: 8.6 ± 0.32 km per night, wild-type: 7.5 ± 0.41 km per night) but no change in the circadian rhythm of usage.

### 3.2. Physical Parameters of 5xFAD Mice under Acute and Chronic Saucer Wheel Usage

**Figure 2:**
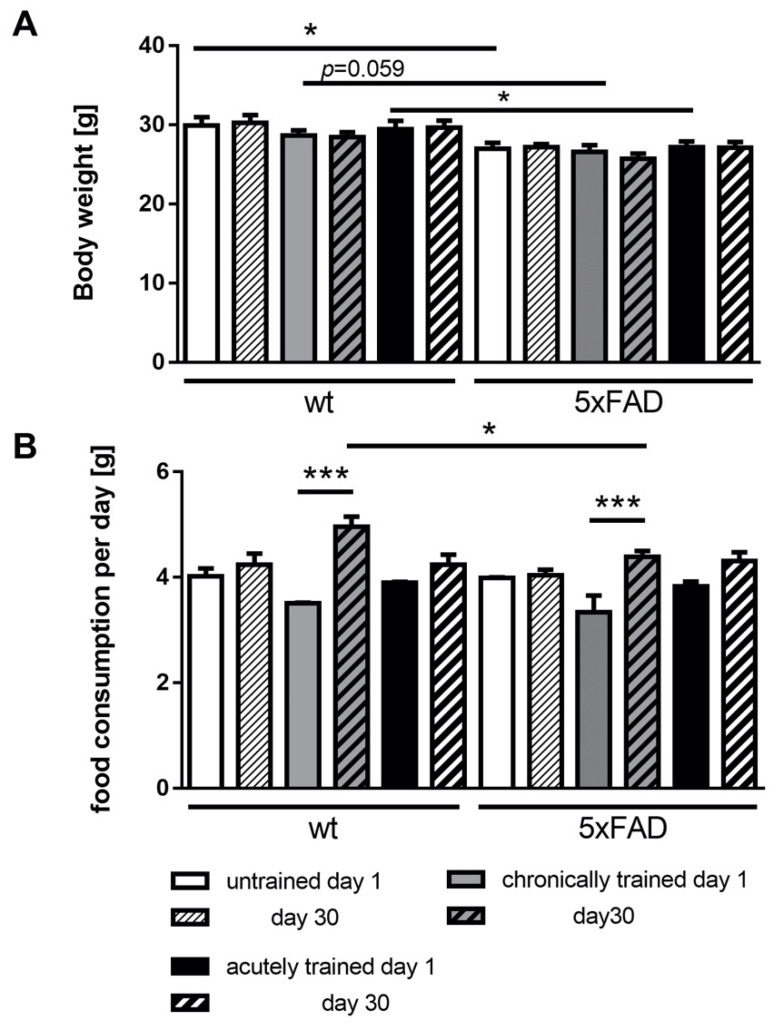
Bodyweight development and food intake of 5xFAD and wild-type mice depending on physical training. (**A**) Body weight was measured at the same time of the day two times weekly. Here, the start weight and the weight at the end of the experiment are indicated. (**B**) Food consumption was measured weekly and is calculated per day. Data are presented as mean + standard error of the mean (SEM) (*n* = 6 for untrained and acutely trained, n = 7 for chronically trained mice). Statistical analysis: ordinary one-way ANOVA with the Fisher’s least significant difference (LSD) test (* *p* < 0.05; *** *p* < 0.001).

Physical activity might affect general parameters such as body weight or feeding behavior (caloric intake) that must be considered. Therefore, body weight was measured twice weekly. As depicted in Figure 2A and reported previously [60], male 5xFAD mice, in general, had a lower body weight as compared to wild-type controls at the age of 2 months but also at the end of the experiment. However, body weight did not change due to the respective activity group. Food consumption was elevated in both chronic training groups independently of the genotype when comparing with the starting point and the end of the experiment (Figure 2B). Interestingly, the increase in food intake was smaller in chronically trained 5xFAD males than in their wild-type littermates. Within the last night, the acute training phase led to a slight non-significant increase in food consumption in both groups.

**Figure 3:**
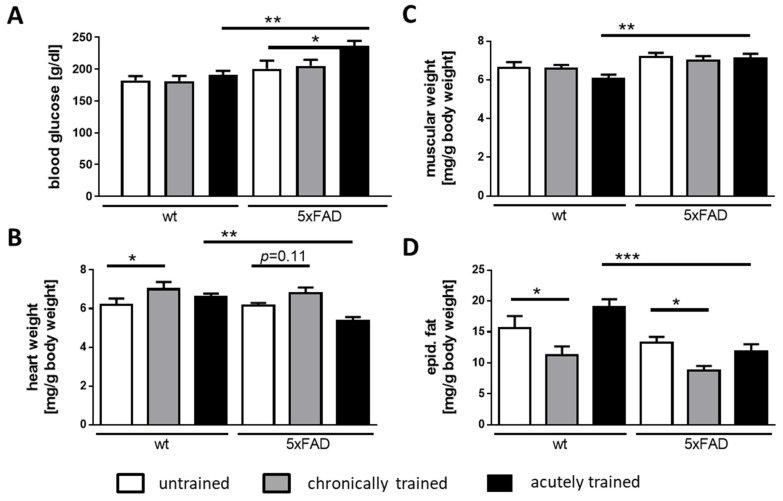
Physical parameters in 5xFAD and wild-type mice after different activity regimens. Upon sacrifice, truncal blood glucose was measured (**A**) and Gastrocnemius of both hind legs (**B**), heart (**C**), and abdominal fat (**D**) were dissected and weighed. Data are presented as mean + SEM (*n* = 6 for untrained and acutely trained, *n* = 7 for chronically trained mice). Statistical analysis: ordinary one-way ANOVA with the Fisher LSD test (* *p* < 0.05; ** *p* < 0.01; *** *p* < 0.001).

Next, we aimed at quantifying parameters that characterize the training effect in the three groups of mice. For this purpose, blood glucose levels were measured from truncal blood upon sacrifice and the weight of the Gastrocnemius muscle, the heart, and abdominal fat mass was determined (Figure 3). Animals of all three groups were sacrificed in an alternating order to prevent circadian effects. As expected, we observed a reduction in fat mass with regular training (as seen in Figure 3D) and an increase in heart mass (Figure 3B). These parameters were comparable for both genotypes even though the heart mass elevation was not statistically significant in transgenic mice.

More astonishing was the effect of acute saucer wheel training on 5xFAD mice: in all examined parameters, the acutely trained group of 5xFAD males differed from their wild-type littermates. For example, the increase in blood glucose levels that was only subtly observable in wild-type mice reached statistical significance in transgenic animals (Figure 3A). The heart mass was reduced in the acutely trained transgenic animals compared to controls (Figure 3B). Moreover, we measured significantly lower abdominal fat mass amounts relative to the wild-type animals after acute training (Figure 3D). All these findings lead to the assumption that 5xFAD mice differ metabolically from their wild-type littermates.

**Figure 4:**
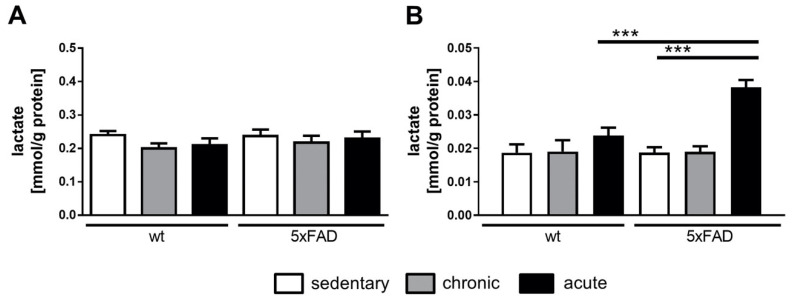
Lactate levels in serum (**A**) and brain tissue (**B**) homogenates of 5xFAD and wild-type mice. Serum and brain homogenate lactate were assessed by an enzymatic assay. Values were normalized to protein content and are presented as mean + SEM (*n* = 6 for untrained and acutely trained, *n* = 7 for chronically trained mice). Statistical analysis: ordinary one-way ANOVA with the Fisher LSD test (*** *p* < 0.001).

An important metabolite for brain homeostasis that has only gained attention within the last few years is lactate. Both genotypes were indistinguishable in terms of serum lactate (Figure 4A). However, the lactate level of brain homogenates was quite striking in the AD mouse model: while chronic activity displayed no effect at all as compared to untrained mice, the acute physical activity for 18 h resulted in doubled lactate levels (Figure 4B), indicating that the physical activity had a direct effect not only on muscle or heart weight but also on brain metabolism.

### 3.3. RNA-Seq Analysis of 5xFAD and Wild-Type Mice with Chronic and Acute Physical Activity

To unravel if the pronounced impact of acute training on 5xFAD mice also corresponded to transcriptomic changes, we employed high-throughput sequencing of RNA extracted from the preserved brain hemispheres of the transgenic and wild-type mice.

**Figure 5:**
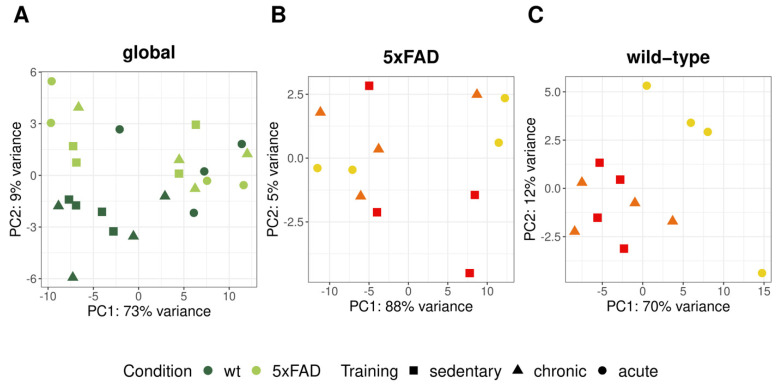
Principal component analysis of normalized RNA counts from 5xFAD and wild-type mice after different training regimens. Using principal component analysis, the highest variances within the gene counts corresponding to principal component 1 (PC1) and principal component 2 (PC2) were calculated and plotted for (**A**) all (*n* = 24), (**B**) 5xFAD (*n* = 12), and (**C**) wild-type (*n* = 12) mice. In (**A**), dark green dots represent wild-type samples, while light green dots correspond to 5xFAD mice. The shapes of the symbols and the colors in (**B**,**C**) show the different training groups (red + circle = acute, orange + triangle = chronic, and yellow + squares = sedentary). The corresponding legend is located on the bottom.

After normalization and filtering of the aligned RNA-seq reads by DESeq2, 20,132 of 24,418 genes returned a non-zero total read count. First, we performed a principal component analysis (PCA) [52] to determine whether the highest variances within the gene counts were related to genotype and training differences. In the PCA, including all samples, principal component 1 (PC1) captured 70% of the variance in the data (Figure 5A). Along PC2, which accounted for 9% of the variance, wild-type mice were partially separated from the 5xFAD mice, indicating genotype-associated differences. Within the 5xFAD mice, the training groups did not cluster together based on the first and second PC and the first PC already captured 90% of the variance (Figure 5B). This indicated that the effect of training on the brain transcriptomic profile was relatively small in comparison to general biological differences in 5xFAD mice. We observed the same trend for untrained and chronically trained wild-type mice, whereas all acutely trained wild-type mice were clearly separated from animals exposed to chronic or no training.

#### 3.3.1. Differential Expression Analysis between Different Physical Activity Levels in 5xFAD and Wild-Type Mice

**Figure 6:**
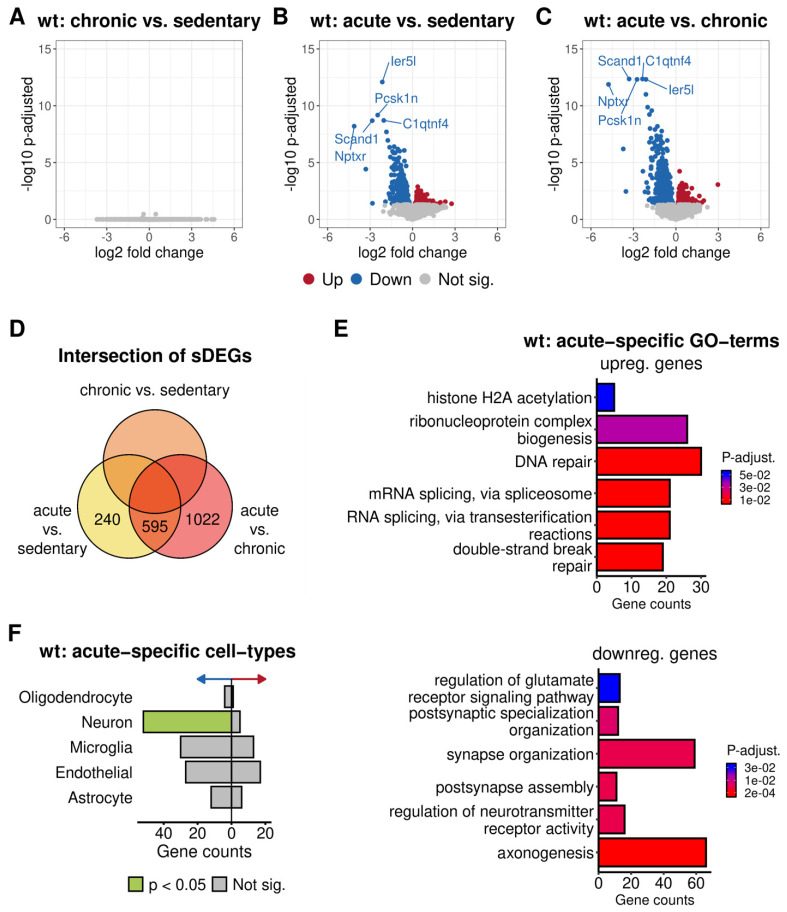
Differential expression analysis results of different physical activity levels among wild-type mice. The volcano plots in (**A**–**C**) visualize the negative log10 of the adjusted *p*-value and the corresponding log2-fold change of the differential expression analyses between (**A**) chronic training and no training (**B**) acute training and no training (**C**) acute training and chronic training of wild-type mice. Each group consisted of four samples. Positive and negative log2-fold change values correspond to up- and downregulation in chronic (**A**) and acute training (**B**) compared to no training and acute training (**C**) compared to chronic training. Differentially expressed genes (DEGs) with an adjusted *p*-value < 0.05 are colored in red or blue corresponding to up- or downregulation, respectively. The five most significant genes are labeled by their gene symbols. All non-significant genes are colored gray. (**D**) shows the intersection of the DEGs found in each comparison. Empty fields represent a zero overlap. In (**E**), the number of genes for significantly enriched Gene Ontology (GO) terms of the acute-specific up- and downregulated genes from the differential expression analysis (DEA) of acute vs. sedentary and acute vs. chronic groups is visualized using bar plots. The color represents the significance of the adjusted *p*-value. (**F**) shows the cell type enrichment results for the acute-training-specific genes. The right and left bars correspond to upregulated and downregulated genes represented by the red and blue arrows’ direction. Green bars correspond to significantly enriched cell-types, while gray represents no significance.

In order to investigate the impact of different levels of physical activity on RNA expression regulation, we compared the transcriptomes of the different activity groups in either 5xFAD or wild-type mice. Interestingly, no transcriptional differences could be detected among the pairwise combinations of sedentary, acutely and chronically trained 5xFAD mice using an adjusted *p*-value (*p*-adj.) < 0.05 except for one gene—Secretogranin-1 (Chgb) (Supplementary Figure S3). Chgb is a neuroendocrine secretory granule protein that was significantly downregulated in 5xFAD mice with one day of wheel running compared to those with no training (*p*-adj. = 0.02, log2 fold change (LFC) = −0.275). Furthermore, the difference was almost statistically significant comparing acutely trained vs. untrained mice (*p*-adj. = 0.093, LFC = −0.258). Among the wild-type mice, the acutely trained animals displayed an altered expression pattern compared to both sedentary and chronically trained mice with 835 and 1617 differentially expressed genes, respectively (DEGs) (Figure 6A–D; Supplementary Table S1). Out of these genes, 595 were commonly up- or downregulated in 4-week trained and untrained mice compared to wild-type animals with one day of wheel running (Figure 6D). These results indicated that acute or persistent physical activity did not alter the basic gene regulation of transgenic mice. In contrast, acute activity was associated with a pronounced effect on gene regulation in wild-type mice.

#### 3.3.2. Functional Annotation of the Altered Gene Expression of Acutely Trained Wild-Type Mice

To uncover the biological background of the transcriptional differences in wild-type mice possibly evoked by one day of voluntary wheel running, we applied the Gene Ontology (GO) term, KEGG pathway, and cell-type enrichment analyses to the significantly up- and downregulated genes that we found in either acute vs. sedentary or acute vs. chronic physical activity. All terms or cells with *p*-adj. < 0.05 were considered as significant based on the statistical overrepresentation test.

The genes that were upregulated in acutely trained wild-type mice were significantly enriched for GO terms mainly involved in Biological Processes of DNA metabolism and transcriptional regulation like “DNA repair” (*p*-adj. = 1.42 × 10^4^), “histone modification” (*p*-adj. = 2.16 × 10^2^), and “mRNA splicing, via spliceosome” (*p*-adj. = 1.22 × 10^4^) (Figure 6E; Supplementary Table S2). On the other hand, many enriched terms of the downregulated genes were involved in general neuronal mechanisms including “axonogenesis” (*p*-adj. = 3.46 × 10^8^), “post-synapse assembly” (*p*-adj. = 9.6 × 10^6^) and “regulation of neurotransmitter receptor activity” (*p*-adj. = 8.42 × 10^6^) (Figure 6E; Supplementary Table S2). Interestingly, the KEGG pathway analysis of all acute-specific genes resulted in a significant enrichment of terms related to neurodegenerative diseases with a *p*-adj. < 0.05. Additionally, the “Spliceosome” pathway was also overrepresented in the acute-specific genes (*p*-adj. = 4.71 × 10^2^) (Supplementary Table S2). The cell-type enrichment analysis supported the GO term, and KEGG pathway results as the acute-specific downregulated genes were significantly enriched for neuronal cells (*p*-adj. = 1.32 × 10^4^) (Figure 6F, Supplementary Table S2). This finding indicated that the short-term wheel running of wild-type mice might induce a neuronal stress state in the brain, altering the homeostasis of neuron functions and possibly overregulating basic DNA and RNA mechanistic processes in parallel.

#### 3.3.3. Differential Expression Analysis between 5xFAD and Wild-Type Mice after Different Physical Activity Levels

**Figure 7:**
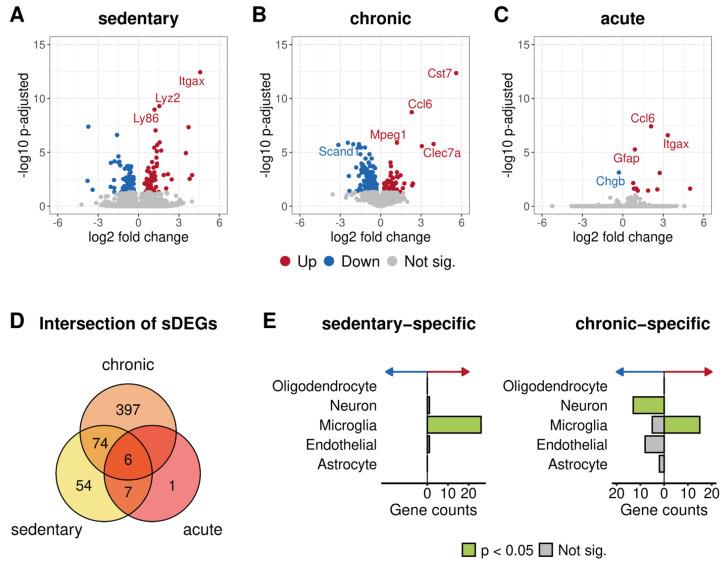
Differential expression analysis results of 5xFAD compared to wild-type mice at different levels of physical activity. The volcano plots in A–C visualize the negative log10 of the adjusted *p*-value and the corresponding log2-fold change of the differential expression analyses between (**A**) sedentary 5xFAD vs. wild-type mice, (**B**) chronically trained 5xFAD vs. wild-type mice, and (**C**) acutely trained 5xFAD vs. wild-type mice. Each group consisted of four samples. Positive and negative log2-fold change values correspond to up- and downregulated genes in 5xFAD mice at the respective level of physical activity. Differentially expressed genes with an adjusted *p*-value < 0.05 are colored in red or blue, corresponding to up- or downregulation. The five most significant genes are labeled by their gene symbols. All non-significant genes are colored gray. (**D**) shows the intersection of the DEGs found in each comparison. Empty fields represent a zero overlap. (**E**) shows the cell type enrichment results for the sedentary-specific (left) and chronic-specific DEGs (right) while the right and left bars correspond to upregulated and downregulated genes represented by the direction of the red and blue arrows. Green bars visualize significantly enriched cell-types, while gray represents no significance.

Next, we investigated the differences in gene expression between 5xFAD and wild-type animals at each training duration level. The DEA between untrained 5xFAD and wild-type mice revealed 141 DEGs (*p*-adj. < 0.05) (Figure 7A; Supplementary Table S3). Since environmental factors did not influence these mice, the detected gene expression differences were likely to be disease-related. Regarding the transcriptional patterns following chronic training, the comparison between the genotypes yielded the most considerable difference in expression with 477 DEGs. Notably, more than 80% of these genes were associated with a significantly lower expression in 5xFAD mice (Figure 7B, Supplementary Table S3). Eighty genes were commonly regulated, comparing 5xFAD with wild-type mice after no training and 30 days of voluntary physical activity (Figure 7D).

Acute training resulted in 14 DEGs between 5xFAD and wild-type mice, of which 13 were upregulated (Figure 7C; Supplementary Table S3). All upregulated genes found after acute activity showed a significant upregulation in 5xFAD mice after chronic and/or no training compared to wild-type animals (Figure 7D). Moreover, nine of these 13 upregulated genes (Cst7, Ccl6, Itgax, Gfap, Clec7a, Ccp4, Cd68, Ccl3, and Trem2) are already known to be differentially expressed in the 5xFAD mouse model [61]. After one day of wheel running, the only downregulated gene was again Chgb, the same gene we previously found to be downregulated when comparing acutely trained with chronically trained 5xFAD mice.

#### 3.3.4. Functional Annotation of Genotype-Specific Genes Found after Chronic and No Training

The genes that were upregulated in the 5xFAD model without any training as compared to wild-type mice were mainly enriched for GO terms of immune-related processes (Supplementary Table S4). The same was true for the upregulated genes found in chronically trained 5xFAD compared to wild-type animals as well as for the shared upregulated genes of the sedentary and chronically trained transgenic vs. wild-type mice (Supplementary Table S4). Moreover, the microglia cell type was significantly enriched in the upregulated genes in 5xFAD compared to wild-type mice after chronic training and especially without training (Figure 7E, Supplementary Table S4).

## 4. Discussion

Our study demonstrated that brain lactate levels increased dramatically after acute voluntary wheel running (1 day) only in 5xFAD mice but not in wild-type animals. In contrast, plasma lactate levels remained unchanged in all animals after physical activity. Interestingly, the whole brain’s subsequent molecular analysis revealed that the transcriptional patterns of 5xFAD mice after acute, chronic, and no training were very similar. In contrast, the acutely trained wild-type mice showed a significantly altered transcriptome compared with both groups either receiving no access to the wheel or 30 days of voluntary wheel running.

### 4.1. Brain Lactate Was Drastically Increased in 5xFAD Mice after Acute Physical Activity

Lactate is a metabolite released from muscle cells during and after intense physical activity [62]. Its conversion from pyruvate by lactate dehydrogenase is facilitated under conditions of reduced oxygen availability, so-called aerobic glycolysis [63,64,65]. As a result, after physical exercise, there is an acute time-restricted increase in lactate levels in circulating blood, which depends on the intensity of physical activity and individual cardiovascular fitness [66,67,68]. In addition to its role in replenishing pyruvate in the liver to provide substrates for gluconeogenesis, lactate is preferentially consumed and metabolized by neurons in an activity-dependent manner [69,70]. Several studies on rodent models have shown that neuronal lactate uptake plays an essential role in long-term memory functions and neurogenesis [71,72]. As an additional fuel of the brain, lactate is proposed as one of the main actors mediating the beneficial effects of physical activity on cognition.

In contrast to our findings, El Hayek and colleagues reported elevated lactate concentrations in healthy mice’s hippocampus after 30 days of voluntary wheel running, which was associated with cognitive improvements [73]. Even though we used the same training regime and the same mouse strain, a possible reason for the non-rising lactate levels after chronic training in our study could be that we measured the lactate concentration in whole-brain hemisphere tissue rather than in a specific region. Moreover, the mice in El Hayek’s study were one month younger than the mice in our analysis. On the one hand, this may indicate that regular physical activity induces region-specific increases in lactate levels, while the brain’s overall concentration remains unchanged. On the other hand, the animals’ age may also play an essential role in lactate enrichment due to physical effort.

Furthermore, we observed increased lactate brain levels in acutely trained 5xFAD animals but not in their wild-type counterparts. Therefore, this finding could indicate an overreaction to a possible neuronal activation and increased energy demands during physical activity in the AD model mice in order to compensate for a disease-driven energy deficit, which is common in AD [74]. The overreaction could be further enhanced by stress or arousal caused by the new motor stimulation of one-day trained animals. Previous studies have shown that rodents exposed to a new environment or behavioral testing exhibit increased aerobic glycolysis leading to higher hippocampal lactate levels and improved memory functions and gene expression changes [64]. Whether this reaction is enhanced under pathological conditions has not been investigated yet.

Furthermore, our results suggest that the lactate increase observed in acutely trained 5xFAD mice was mainly attributable to synthesis within the brain rather than transport from the blood. One possible lactate production mechanism within the brain is associated with the astrocyte-neuron-lactate shuttle (ANLS). This model hypothesizes that neurons release glutamate during neuronal activation, transport it to astrocytes that generate lactate, which is then transported back to neurons and used as an energy source for neuronal functions [64]. In line with this, Zuend and colleagues recently reported that lactate is released by astrocytes and taken up by neurons due to acute environmental arousal in mice [75]. In contrast, Díaz-García and colleagues provided evidence that acutely excited neurons perform aerobic glycolysis by themselves rather than taking up lactate produced by astrocytes [76,77]. Unfortunately, our experimental design does not allow us to ascertain if the lactate source in our study was attributable to astrocyte or direct neuronal production.

### 4.2. One Day of Voluntary Wheel Running Altered Transcription Only in Wild-Type but Not in 5xFAD Mice

Multiple studies investigating acute or chronic training were able to show beneficial effects on cognition, Aβ plaques, brain size, immune functions, or expression of neurotrophins in different transgenic mouse models of Alzheimer’s disease [18,22,23,24]. However, other studies could not confirm the positive influence of physical exercise on AD [35,36,37,38,39]. Recently, the 5xFAD mouse model was investigated after chronic voluntary wheel training, and neither cognitive improvements nor decreased Aβ levels and no reduction in AD-driven neuroinflammation were detected [37,38]. This is in accordance with our findings at the transcriptome level. One month of voluntary wheel training did not change the global gene expression in 5xFAD mice compared to sedentary animals. However, as we did not consider behavior or immune-related functions, we cannot conclude that regular activity cannot improve the pathology of the 5xFAD mouse model. Furthermore, other biological layers like the metabolome or proteome could be positively influenced by acute and chronic activity, while the transcriptome might play a minor role.

Additionally, the secretory-related gene Chgb was significantly downregulated in acutely trained 5xFAD mice compared to chronically trained and sedentary transgenic mice. The deposition of Aβ has previously been reported to be related to the downregulation of genes involved in the secretory pathway, including Chgb [78]. The slight reduction in Chgb expression potentially indicates that the short-term activity might enhance the AD-like decline by downregulating important secretory pathway factors. However, this finding contradicts the potentially beneficial impact of the lactate increases on the neuronal function within the acutely trained 5xFAD group. As physical activity is suggested to be more of a preventive strategy against cognitive decline, it is possible that the disease phenotype of the aggressive 5xFAD mouse model is already too advanced at two months of age to be alleviated by an external intervention such as physical activity.

Regarding the physical activity duration in wild-type mice, only one day of voluntary wheel running altered the transcriptional pattern as compared to sedentary mice. In humans, one single bout of exercise is suggested to positively influence cognition as well as mood and emotional state to a small extent [34]. Several studies investigating neurochemical measurements after one exercise session have shown that specific neurotransmitters, neurotrophins, and other essential metabolites related to neurogenesis and brain plasticity are enhanced after this short time [34]. In our study, DEA revealed that axons’ growth and development within acutely trained wild-type mice were likely inhibited. Many under-expressed genes were associated with axonogenesis assuming that these genes are positively correlated to this process. However, human studies have also reported negative effects after acute exercise [34,79]. For instance, acute stress and acute exercise share several mechanisms like the activation of the hypothalamic–pituitary–adrenal (HPA) axis and the sympathetic nervous system, both related to immunity.

In contrast to regular physical activity, acute exercise has been shown to reduce immunity and increase susceptibility to infections [80]. Furthermore, elevated oxidative stress levels have been correlated with acute exercise, which is involved in functional alterations, especially in the central nervous system [79]. Thus, changes in the transcriptome of the acutely trained wild-type mice in our study might correspond to a transient response to acute stress. In contrast, regular physical activity leads to an adaptation and a return of the transcriptome to a homeostatic state.

### 4.3. Immune-Related Genes Were Upregulated in Untrained and Chronically Trained 5xFAD Mice Compared to Their Wild-Type Counterparts

Furthermore, we investigated activity-genotype specific transcriptional regulation by comparing 5xFAD with wild-type mice after one day, 30 days, or no voluntary wheel training. Importantly, sedentary and chronically trained animals revealed several disease-driven gene expression differences. Here, we could demonstrate that the over-expressed genes in chronically trained or untrained 5xFAD mice compared to wild-type littermates were mainly involved in immune response processes. Neuroinflammation is one of the significant hallmarks of AD and has recently been suggested to be the main driver of the early progression of AD [81]. Within the 5xFAD model, immune-system changes are likely to occur prior to plaque deposition development in 2-month-old 5xFAD mice [82]. In addition, we found that the genes upregulated in 5xFAD mice were significantly associated with microglial cells. Microglia are responsible for activating the brain’s innate immune system, and their accumulation contributes to neurodegenerative pathologies [40,83,84]. Interestingly, the comparison of the genotypes after regular training yielded a much larger transcriptional gap between 5xFAD and wild-type animals than in sedentary mice, and the majority of genes were downregulated. However, no biological function was overrepresented for these under-expressed genes.

In contrast, acutely trained wild-type mice, which differed from the other wild-type groups, had a similar transcriptomic profile to their 5xFAD counterparts. In agreement with our findings, a human study showed that the human ortholog of Chgb, the only downregulated gene in acutely trained 5xFAD compared to wild-type mice, was significantly downregulated in AD patients compared to healthy controls [85]. Notably, many immune-response-associated genes were no longer differentially regulated between the transgenic and wild-type mice, as was the case for the chronically trained or sedentary animals. These findings corroborate the assumption that acute physical activity triggered a stress response in wild-type animals, which shifted their transcriptome profile in the direction of their transgenic counterparts.

Taken together, our results suggest that the brain’s global transcriptome might not be the primary mediator of the beneficial effects found in healthy and AD individuals due to regular physical activity. Interestingly, acute training was associated with drastic changes in both 5xFAD and wild-type mice, however, in entirely different ways. Brain lactate concentrations and other physiological parameters were altered in 5xFAD mice. In contrast, the brain transcriptome of wild-type animals changed its expression patterns after acute training. These genotype-specific differences should be further investigated in order to elucidate the real link between acute physical activity and AD.

## Data Availability

The raw RNA-seq data and the corresponding feature count table discussed in this publication have been deposited in NCBI’s Gene Expression Omnibus [86] and are accessible through GEO Series accession number GSE164798 (https://www.ncbi.nlm.nih.gov/geo/query/acc.cgi?acc=GSE164798).

## Supplementary Materials

The following are available online at https://www.mdpi.com/2073-4409/10/3/693/s1, Figure S1: Normalized gene counts of mutated and mutation-correlated genes that produce the 5xFAD mouse model, Figure S2: Principal Component Analysis of normalized counts from training-specific groups, Figure S3: Volcano plots of all pairwise contrasts of training groups in 5xFAD samples. Table S1: Differential expression results from the pairwise comparisons between acute and chronic training and between acute and no training of wild-type mice. Table S2: Functional annotation results of differential expressed genes observed from the comparisons of different activity regimens in wild-type mice, Table S3: Differential expression results from the pairwise comparisons between 5xFAD and wild-type mice after no training, chronic training and acute training, Table S4: Functional annotation results of differential expressed genes observed for the comparisons between 5xFAD and wild-type mice after each training duration.
