# Supplementary material for: Voluntary Wheel Running Did Not Alter Gene Expression in 5xfad Mice, but in Wild-Type Animals Exclusively after One-Day of Physical Activity
Source: Cells. 2021 Mar 20;10(3):693. doi: 10.3390/cells10030693 (PMC8004053; doi:10.3390/cells10030693)
Supplement: Supplementary file 1 [file cells-10-00693-s001.zip › Supplementary_Material/Supplementary_Figures_Wierczeiko.docx]

Supplementary Figures

“Voluntary wheel running did not alter gene expression in 5xFAD mice, but in wild-type animals exclusively after one-day exercise bout”


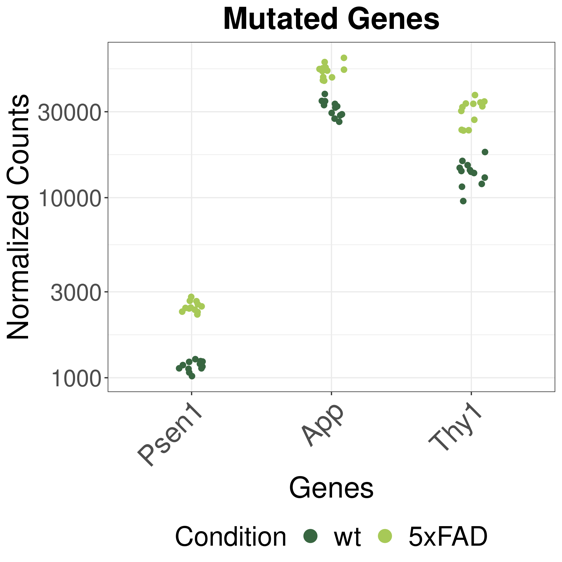


**Figure S1: Normalized gene counts of mutated and mutation-correlated genes that produce the 5xFAD mouse model.** The raw gene counts of Psen1, App, and Thy1 were normalized within the global expression table using the size factor provided by DESeq2. Dark green dots represent the wild-type samples, and the 5xFAD mice are colored in light green. The counts of all three genes show two clusters that separate the disease model from healthy controls, thus demonstrating successful genotyping at the transcriptional level.


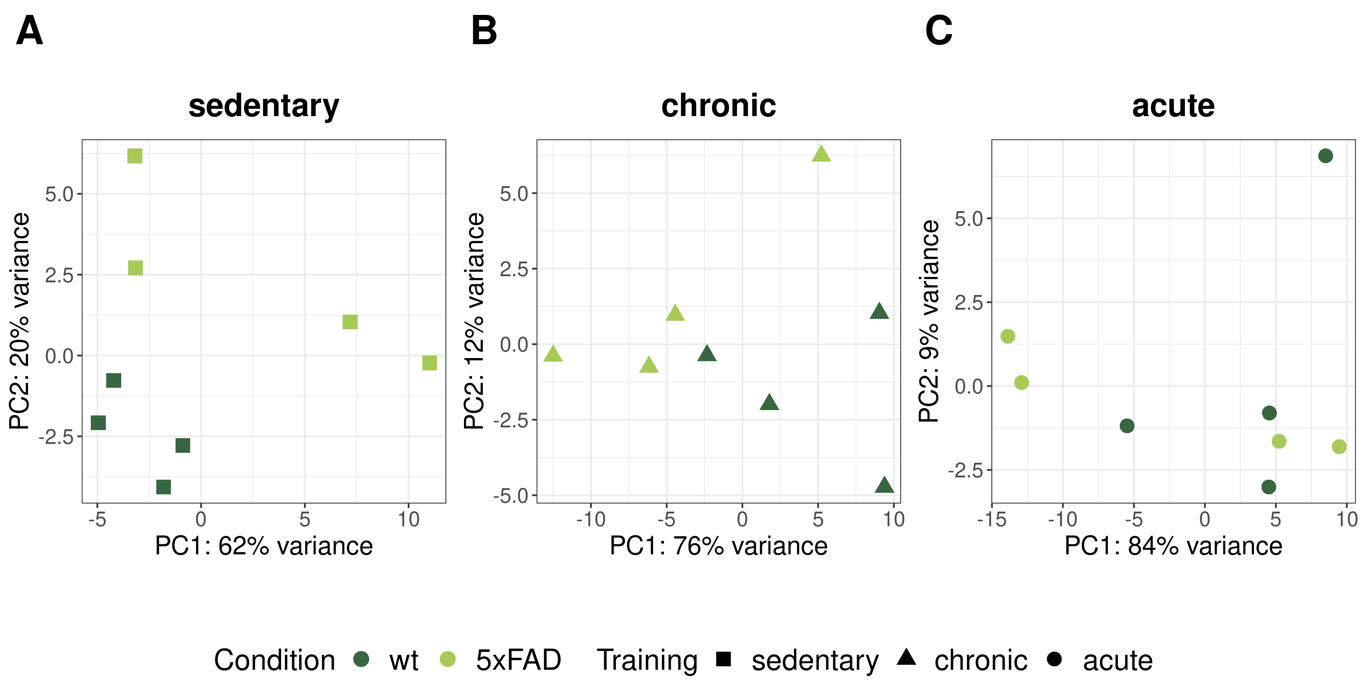


**Figure S2: Principal Component Analysis of normalized counts from training-specific groups.** Using principal component analysis, the highest variances within the gene counts corresponding to principal component 1 (PC1) and principal component 2 (PC2) were calculated and plotted for A) sedentary (n = 8), B) chronically trained (n = 8) and C) wild-type (n = 8) mice. In A), dark green dots represent wild-type samples, while light green dots correspond to 5xFAD mice. The shapes of the symbols and the colors show the different training groups. The corresponding legend is located on the bottom.


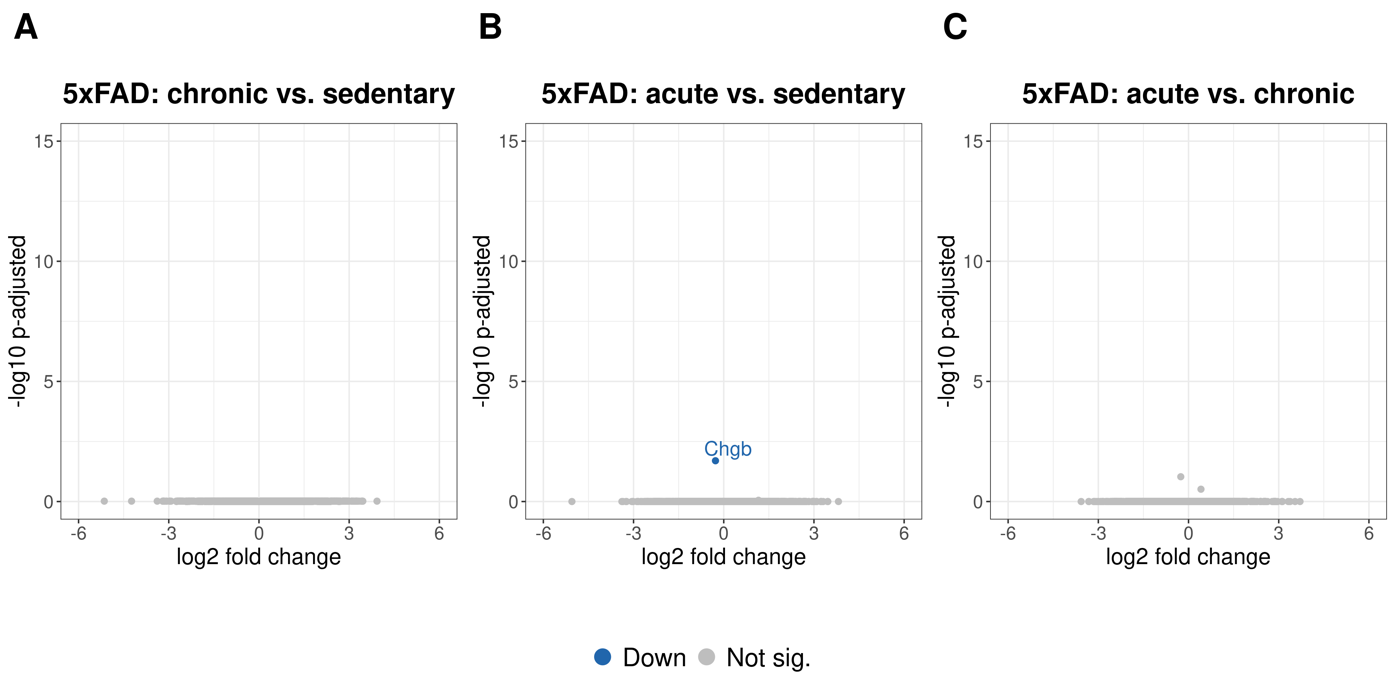


**Figure S3: Volcano plots of all pairwise contrasts of training groups in 5xFAD samples.** The volcano plots visualize the negative log10 of the adjusted p-value and the corresponding log2-fold change of the differential expression analysis between A) chronic training and sedentariness (n=3+3), B) acute training, and sedentariness and C) acute training and chronic training of 5xFAD mice. Each group consisted of four samples. Positive and negative log2-fold change values correspond to up- and downregulation in each comparison. Significant differentially expressed genes with an adjusted p-value < 0.05 are colored in blue, corresponding to upregulation. All non-significant genes are colored gray (see legend).
